# Modes of arrival, door to balloon time and its impact on morbidity and mortality for ST elevation myocardial infarct

**DOI:** 10.1186/cc10790

**Published:** 2012-03-20

**Authors:** YC Chia

**Affiliations:** 1 Tan Tock Seng Hospital, Singapore

## Introduction

Timely reperfusion of the occluded coronary artery is crucial in reducing the amount of myocardial damage in patients with ST elevation myocardial infarct (STEMI). This study aims to examine the common presenting symptoms of patients with STEMI, their modes of arrival at the emergency department (ED) and its impact on door-to-balloon (D2B) time and in-hospital morbidity and mortality.

## Methods

In this retrospective study, the medical records of 619 patients with an admitting diagnosis of STEMI from Tan Tock Seng Hospital, Emergency Department between 1 January 2009 and 31 December 2009 were reviewed. We extracted data from the electronic records of the emergency case notes and inpatient discharge summaries.

## Results

Among 619 patients, 363 (58.6%) arrived by emergency medical services (EMS) and 256 (41.4%) by self-transport. Three hundred and thirty (53.3%) patients underwent emergency angiography, of which 313 (94.9%) were treated with percutaneous coronary intervention (PCI), eight (2.4%) with coronary artery bypass grafting (CABG) and nine (2.7%) were conservatively managed. The D2B time was significantly shorter in patients who arrived by EMS (60 vs. 82 minutes; *P *< 0.001). There was no difference in D2B time between patients who arrived in the day (06:00 to 17:59 hours) or at night (18:00 to 05:59 hours). Chest pain, shortness of breath and diaphoresis were the three commonest presenting symptoms in patients with STEMI regardless of their mode of arrival. Previous myocardial infarction, PCI or CABG did not influence the mode of transport. Patients who arrived by EMS had a higher incidence of cardiogenic shock (20.7% vs. 11.7%; *P *= 0.020) and were significantly older (63 vs. 59 years; *P *= 0.004) than patients who arrived by self-transport. Patients who arrived by EMS had a higher in-hospital mortality rate (12.1% vs. 5.1%; *P *= 0.003) and a longer mean length of stay compared to those who arrived by self-transport (6 vs. 4 days; *P *= 0.004).

## Conclusion

In our study population, patients with STEMI who used EMS tend to be older and arrived in cardiogenic shock. They therefore had a higher incidence of in-hospital mortality and morbidity although their D2B time was shorter compared to those who arrived by self-transport.

